# Progress in Surgery

**Published:** 1898-07-09

**Authors:** 


### Progress in Surgery.

### SURGERY OF THE LIVER AND GALL-BLADDER.

**Cholelithiasis.**—Wendel[1] relates a case of floating gall-bladder and kidney complicated by cholelithiasis with perforation of the gall-bladder. The mobility of the gall-bladder was aided by the great length of the cystic duct, which, although much twisted, had not caused gangrene of the gall-bladder. The patient experienced no discomfort until an infective inflammation occurred in the gall-bladder. The gall-bladder was removed, and the patient made a good recovery.

**Cystic Dilatation of the Common Bile Duct** in a child, aged eight years, is recorded by Russell.[2] This unique condition is regarded by the author as probably being of congenital origin. After cœliotomy, the opening in the cyst was stitched to the abdominal wall, but the patient died four days after the operation from hæmorrhage, the result of uncontrollable oozing from the stitches and into the cyst.

**Traumatic Rupture of the Liver,** followed by the formation of a cystic swelling containing bile-stained fluid, is recorded by Whipple.[3] It seems probable that a bile-duct was torn across at the time of the injury, and that bile had collected underneath the peritoneum covering the liver, and had subsequently separated the layers of the falciform ligament. The swelling was incised and drained, and recovery ensued in this unique case.

**Accessory Lobe of Liver.**—Martin[4] has successfully removed a large pedunculated lobe of the liver, which, together with the gall-bladder, weighed 3¾ lbs. The tumour had a distinctly greenish hue, contrasting markedly with the chocolate-brown colour of the liver. The pedicle, which was about three inches long and four inches broad, was seen to be composed of the cystic duct, of numerous large arteries and veins, and some fibrous tissue, the whole being surrounded by peritoneum. There was no continuity of the glandular substance of the liver with the substance of the tumour. The cystic duct was ligatured and divided, and the rest of the pedicle secured with three interlocking ligatures of stout silk. The whole mass with the gall-bladder was then cut away. When the pedicle was divided it shrank upwards and backwards towards the portal fissure. The liver proper was apparently normally shaped. The round ligament, the suspensory ligament, and the lateral ligaments had no connection with the tumour.

**Hepatoptosis or Floating Liver** is described by Terrier and Auvray.[5] It is commonly found in connection with floating kidney, internal ptosis, uterine displacements, herniæ, and varicoceles. Partial hepatoptosis—such as may arise from the dragging of a tumour—is common. Complete hepatoptosis is rather uncommon, and occurs oftener in women than in men, but Glenard found 25 per cent. of cases in men to 15 per cent. in women. The affection may be congenital or acquired. Congenital cases are due to the absence of the coronary and suspensory ligaments. Most commonly it is acquired. The determining causes are mainly mechanical. Among these are repeated strains, heavy work, violent exercise, severe forced expiration, &c. The influence of corsets is doubtful. The pendulous condition of the belly wall which may follow pregnancy is a fruitful cause of floating liver. Other causes worthy of mention are increase in weight of the liver, atrophy of the connective tissues uniting that organ to the diaphragm. Treatment of partial hepatoptosis has been carried out in three ways: (1) By resection of the "floating lobe"; (2) by fixation to the abdominal wall—*i.e.*, partial hepatopexy; (3) by cholecystostomy, in the hope of indirectly diminishing the lobe. Treatment of complete hepatoptosis may be medical or surgical; the former ought always to be tried first. Disorders of nutrition play an important *rôle*, and therefore call for treatment. A course of tonics and hydrotherapy is advisable. The use of electricity has been advised to increase the tone of the abdominal muscles. Special bandages and binders, with supporting pads, have been employed to sustain the displaced organ. When such treatment fails, one may advise, in a small number of cases, recourse to surgical intervention. The operation of complete hepatopexy has been performed fifteen times. Fixation of the liver is attained by suturing it with catgut to the right costal margin or the abdominal wall. Lucas-Championnière modifies the operation by passing some of the threads through the suspensory ligament. To assure the formation of adhesions it is well to scarify or irritate the surfaces to be approximated. Of eleven cases there were eight recoveries, one death, two cases uncertain. The only death was due to acute peritonitis. The symptoms are thus described by Gamgee[6]: Total hepatoptosis — I. When the descent is due to some great muscular effort, and so is sudden: (1) A twisting pain in the right side; (2) syncope and vomiting; (3) physical signs as detailed below. II. In chronic cases: (1) Pain in abdomen, relieved by rest in bed; (2) sometimes crises associated with jaundice, and resembling biliary colic; (3) pain behind manubrium sterni, due to the connection of the liver with the diaphragm, the diaphragm with the pericardium, and the pericardium with the deep cervical fascia; (4) physical signs: (*a*) Abdominal wall lax and overhanging; (*b*) absence of liver dulness from its normal position; (*c*) dulness in abnormal position, unless intestines have floated between liver and parietes; (*d*) in position of abnormal dulness tumour can be felt, having outline of liver and being movable (unless fixed by adhesions). Partial hepatoptosis: (1) Abdominal pain, severe, generally relieved by rest in bed; (2) vomiting, palpitations, &c; (3) physical signs: (*a*) Clearly defined movable tumour, dull area over which is continuous with liver dulness. Sometimes it can be made out that the tumour is moored in neighbourhood of liver; (*b*) tumour shows a certain degree of ballottement, and moves with the diaphragm.

**Hepatic Abscess.**—Johnston[7] says the most constant and therefore the most valuable symptoms of this affection may be summarised as follows: (1) A history in which dysentery and chill appear; (2) general malaise pronounced; (3) pain and tenderness over liver; (4) enlargement of liver; (5) hectic, sweats and rigors; (6) right side posture; (7) erratic temperature running from 96·5 deg. to 103·5 deg. F.; (8) progressive emaciation; (9) gastric disturbances. In the treatment of this malady no measure will succeed which does not completely evacuate the abscess cavity and allow free drainage. This can only be done with precision and safety by incision. Aspiration, puncture with trocar, opening by caustics and thermo-cautery are uncertain, dangerous, and unsurgical, and should be condemned. The route by which the liver is entered may be either transperitoneal or transpleural—the latter being more difficult. The line of incision must be determined by the position of the abscess. The results of treatment were as follows: In the first eleven cases (1878—1884), which were treated by aspiration, ten died, one recovered. The recovery in this case was brought about by a spontaneous discharge of the abscess through the bronchi. In the second group of seven cases two declined operation. Both died. Five were treated by incision and drainage. Four recovered, one died.

**Hydatids of the Liver.**—Bobroff[8] gives the history of four cases of hydatids of the liver and one case of hydatid cyst of the pancreas successfully operated upon by him. His method of operating differs from that adopted by other surgeons. After incising the cyst and letting out the fluid, he removes the chitinous wall, and carefully disinfects the interior of the fibrous capsule. The sac and abdominal wall are then completely closed by sutures, no drainage being provided.

Convalescence in all five cases was rapid, the patients being able to leave their beds in two or three weeks after the operation. Waring,[9] in his recent Erasmus Wilson lectures, says he has notes of twenty-one cases of hydatid disease of the liver treated by an abdominal operation. Of these sixteen recovered and five terminated fatally. Two of the fatal cases were suppurating at the time of operation, and in one death did not occur till four years afterwards, when suppuration occurred in a sinus which had remained not closed. One was treated by a thoracic operation with a fatal result. Five cases were treated by aspiration. Three of these were afterwards treated by an abdominal incision, and in the others the swelling still remained, but they escaped from observation, and the final result was not known. According to Thomas the mortality in cases which have been treated by the one-stage abdominal operation has been 10·29 per cent., and in those treated by the thoracic operation 29·4 per cent.

**Tumours of the Liver.**—These are generally best treated by resection. The clinical conditions which may be considered to indicate the performance of a surgical operation for the removal of a portion of the liver are, says Waring,[10] as follows: (1) Carcinoma of the gall-bladder, in which the disease has not extended beyond the gall-bladder and the adjacent portion of the liver, and there are no signs of secondary growths either in other parts of the liver or in other viscera. (2) The presence of a localised non-malignant tumour of the liver, such as angioma, angio-fibroma, fibroma, or adenoma, which is increasing in size and is giving rise to trouble. (3) The presence of a localised hepatic tumour which is due to a mass of echinococcus multilocularis. (4) Congenital hernia of a portion of the liver in which the herniated portion cannot be returned within the abdomen. (5) Primary sarcoma of the liver occasionally occurs, and has been treated by excision. (6) Primary carcinoma of the liver is exceedingly rare. If it is in the form of a localised tumour an attempt may be made to remove it. Of fifty-nine cases of removal of portions of the liver collected by Keen, forty-nine recovered, nine terminated fatally, and in one the result was not known. The most important methods which have been practised for the removal of tumours of the liver are the following: (1) The elastic ligature, applied after the liver has been fixed in the abdominal wound. (2) By fixation of the affected part of the liver in the abdominal wound and removal at a later (three to five days) period. It may be removed either with a knife or with a Paquelin's cautery by cutting through the base of the tumour. (3) Small tumours may be treated by dissection of the tumour or cyst from the liver, suture of the hepatic wound, and closure of the parietal wound. (4) By excision of the diseased portion of the liver by a "wedge"-shaped incision. After the diseased portion is exposed, the circulation in the hepatic artery and portal vein is controlled by the application of pressure to the gastro-hepatic omentum. A wedge-shaped piece of the liver, including the diseased portion, is then removed, divided blood vessels ligatured, and the margins of the hepatic incision are then brought into apposition, and fixed by sutures. Waring[11] says that as the result of his experiments on animals he considers this last method the best and safest. As a further result of these experiments he concludes: (1) That the dangers of hæmorrhage after removal of portions of the liver have been much exaggerated. (2) That the best method of arresting and controlling hæmorrhage from liver tissue after it has been incised or torn is by temporary compression of the blood vessels in the gastro-hepatic omentum, and then ligature of the divided vessels. (3) That when the liver is the seat of growths such as mentioned above a surgeon ought to be prepared to carry out an operation for their removal. (4) That the mortality which will result from operations of this kind is not greater than in many of the recognised abdominal operations, such as resection of the intestine.

[1] Annals of Surgery, Feb., 1898, p. 199. [2] Ibid., Dec., 1897, p. 692. [3] Lancet, March 12, 1898. [4] Birm. Med. Rec., Feb., 1898, p. 92. [5] Annals of Surgery, Jan. 1898, p. 115. [6] Birm. Med. Rec., Feb., 1898, p. 10[illegible]. [7] Annals of Surgery, Oct., 1897. [8] Brit. Med. Jour. Epit., Mar. 19, 1898; and Khirurgyra, Jan., 1898. [9] Lancet, Mar. 12, 1898, p. 701, [10] Ibid., Mar. 19, 1898, p. 778. [11] Loc. cit.

